# Multi-Scale Investigation on the Hydration and Microstructure of Portland Cement Blended with Inorganic Softening Sludge

**DOI:** 10.3390/ma19214447

**Published:** 2026-10-22

**Authors:** Zhengyang Qu, Yuanye Chen, Meng Su, Nan Ding, Meiqing Chen, Zhenyun Cheng, Bo Fu

**Affiliations:** School of Hydraulic Engineering and Smart Cities, North Minzu University, Yinchuan 750021, China; 20257673@stu.nmu.edu.cn (Z.Q.); 20257672@stu.nmu.edu.cn (Y.C.); 20247578@stu.nmu.edu.cn (M.S.);

**Keywords:** inorganic softened sludge, ordinary Portland cement, portlandite, hemicarboaluminate, hydration mechanism

## Abstract

Inorganic Softened sludge is a solid by-product generated during water treatment. Owing to its fine particle size and high calcium content, ISS has potential for use as a mineral admixture in cement-based materials. However, its specific role and underlying mechanisms in cementitious materials remain unclear. Therefore, this study systematically investigates the effects of different ISS replacement levels on the workability, mechanical properties, and hydration evolution of cement paste. The results showed that increasing the ISS replacement level progressively reduced the flowability of the paste and prolonged the setting time. Compared with the neat cement control, low ISS replacement levels (5–10%) increased the 3 days compressive strength, while causing only a slight reduction in the 28 days compressive strength; in contrast, high ISS replacement levels (15–30%) resulted in a pronounced decrease in compressive strength. Particle-size analysis showed that ISS had a smaller median particle size than OPC, suggesting a potential physical filling effect. Microstructural analyses indicated that ISS incorporation did not appreciably alter the types of major hydration products, while the detection of hemicarboaluminate (Hc) suggested that carbonate introduced by ISS may have participated in reactions involving aluminate phases. BSE/EDS observations revealed residual ISS-associated particles within the hydrated matrix, with Ca- and Si-enriched regions adjacent to some particles, consistent with the elemental composition of C-S-H-like hydration products. At low replacement levels, particle surface effects and possible physical filling effects may jointly contribute to the observed strength changes, potentially enhancing the early-age strength of the cementitious matrix.

## 1. Introduction

Ordinary Portland cement (OPC), one of the most widely used cementitious materials in modern construction, is associated with substantial energy consumption and CO_2_ emissions during its production, posing a significant challenge to global carbon-reduction targets [1]. The cement industry is estimated to account for approximately 7–8% of global anthropogenic CO_2_ emissions [2]. Therefore, reducing clinker consumption and developing low-carbon alternative cementitious materials have become important strategies for promoting the sustainability of construction materials. In recent years, a wide range of industrial solid wastes has been increasingly utilized to produce supplementary cementitious materials (SCMs), which can partially replace OPC and reduce the associated environmental burden. Among them, conventional SCMs, such as fly ash (FA), ground granulated blast-furnace slag (GBFS), and silica fume (SF), have been extensively incorporated into cement-based materials owing to their potential pozzolanic activity or latent hydraulic reactivity [3,4,5]. However, the availability of high-quality industrial by-products has become increasingly uncertain due to the gradual depletion of readily available resources and ongoing changes in energy structures. Consequently, the development of novel alternative materials with stable availability, low cost, and environmental compatibility has emerged as an important pathway toward the green transformation of the cement industry.

In recent years, solid by-products generated from municipal and industrial processes have attracted increasing attention owing to their abundant availability and relatively low recycling rates. Among these materials, inorganic softened sludge (ISS), a solid by-product generated in water treatment processes, has gradually emerged as a potential resource for novel construction materials. With the growing global demand for potable water and increasingly stringent water quality standards, water softening processes have been widely implemented in water treatment systems, generating substantial quantities of sludge by-products rich in carbonate minerals [6]. At present, ISS is still predominantly managed through landfilling or stockpiling. Although its beneficial utilization in applications such as acidic-soil amendment and construction materials has attracted increasing attention, these reuse pathways remain relatively limited [7,8]. During long-term disposal, some trace constituents accumulated in the sludge may be released under rainwater infiltration and changing environmental conditions and subsequently migrate into surrounding soils and groundwater, thereby posing potential risks of secondary environmental contamination [9,10]. ISS primarily consists of carbonate minerals, and its chemical composition exhibits certain similarities to that of conventional carbonate-based mineral admixtures, such as limestone powder (LS). Previous studies have demonstrated that carbonate-based mineral admixtures can improve particle packing through a microfilling effect and promote the formation of stable carbonate-aluminate hydrates through interactions between carbonate species and aluminate phases, thereby influencing the hydration process and microstructural evolution of cementitious systems [11,12]. Incorporating ISS as a supplementary cementitious material into cement-based systems can not only alleviate the pressure associated with solid waste disposal but also reduce cement clinker consumption, thereby contributing to both the valorization of solid waste and the development of low-carbon construction materials.

ISS differs from natural limestone powder in its origin and may also exhibit different particle morphologies and surface characteristics. Flocculation, sedimentation, and dewatering during water treatment may give rise to characteristic agglomeration patterns, particle morphologies, and surface features in ISS [13]. Although ISS shares compositional similarities with limestone powder and other carbonate-based mineral additions, these similarities do not necessarily imply equivalent performance in cementitious systems, as the influence of particle characteristics must also be considered. A systematic understanding of the effects of ISS replacement level on fresh properties, strength development, and hydration product distribution, together with their relationship to particle characteristics, remains limited. Accordingly, this study investigates dried and ground ISS as a partial replacement for Portland cement at replacement levels of 0–30%, systematically evaluating changes in paste flowability, setting time, and compressive strength. Particle characteristics, phase analysis, and microstructural observations are considered together to discuss possible explanations for the observed performance changes, with particular emphasis on carbonate participation in hydrate phase evolution and the effects of reduced cement content. The findings provide an experimental basis for assessing the performance and limitations of ISS from the investigated source as a cement replacement material.

## 2. Materials and Experimental Methods

### 2.1. Raw Materials

The chemical compositions of the raw materials were determined using X-ray fluorescence (XRF, Thermo Fisher Scientific, Waltham, MA, USA) spectroscopy, and the results are presented in Table 1. Ordinary Portland cement was used to prepare all specimens in this study, and its relevant properties complied with the requirements of GB 175-2023 [14]. The ISS used in this study was collected from the water treatment plant of Ningxia Coal Industry Co., Ltd., China Energy Investment Corporation, Ningxia, China. The plant employs a chemical softening process combining lime–soda ash precipitation with high-density clarification, in which Ca^2+^ and Mg^2+^ are mainly removed as CaCO_3_ and Mg(OH)_2_, respectively. The resulting precipitated sludge was collected as ISS [13]. XRF analysis revealed that the major oxide components of ISS were CaO, SiO_2_, and MgO, collectively accounting for 90.971% of the normalized composition. In addition, minor elements, including Cl, Cu, and Zn, which may influence cement hydration, were detected at mass fractions of 0.315%, 0.0182%, and 0.0252%, respectively. Ordinary tap water was used for mixing.

**Figure 1:**
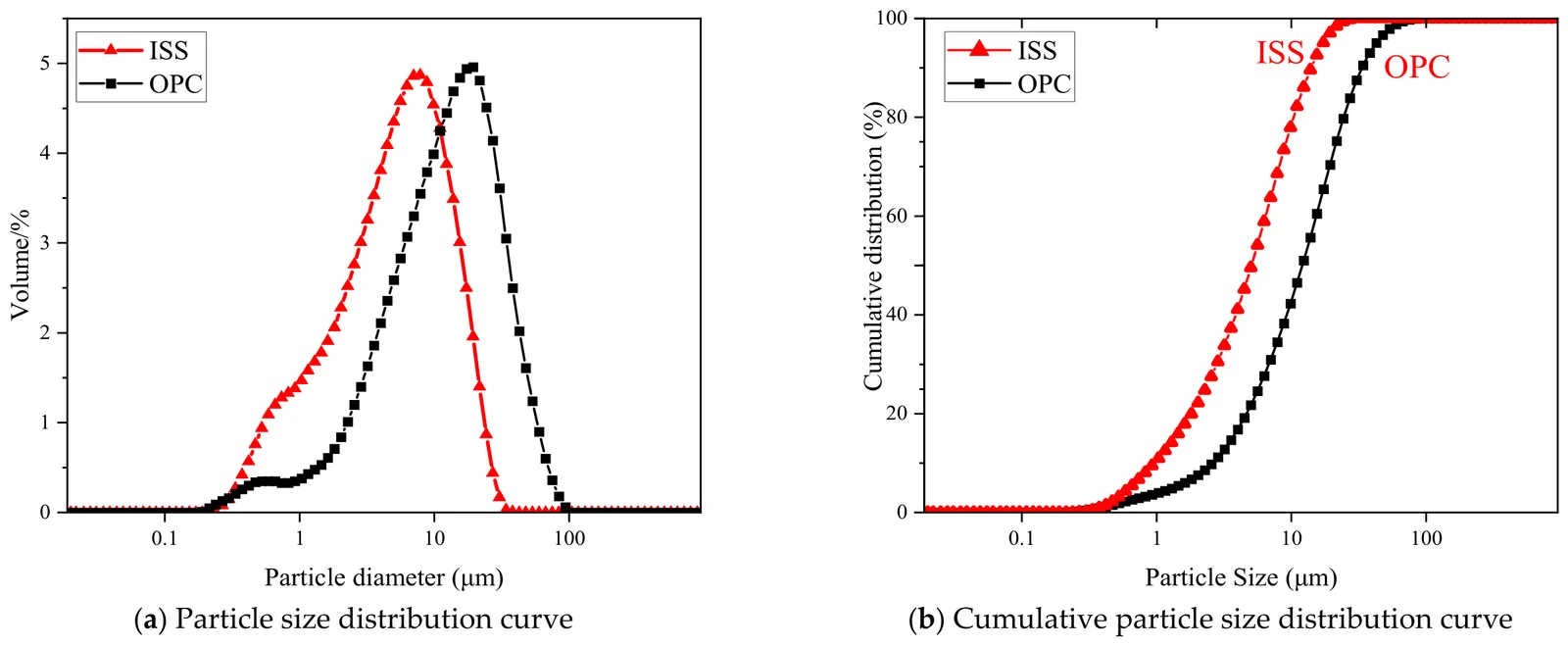
Particle size distribution of inorganic softened sludge and ordinary Portland cement.

The particle size distribution of ISS was determined using a laser particle size analyzer (Bettersize 2000, Dandong Specific Surface Area Instrument Co., Ltd., Dandong, China). Figure 1 presents the particle size distributions of ISS and OPC. Among the powdered raw materials, OPC exhibited a median particle size (D50) of 13.46 μm, whereas ISS had a substantially smaller (D50) of 5.663 μm. The specific surface area measurements were highly consistent with the particle size results. The Specific Surface Area of OPC was 0.419 m^2^/g, while that of ISS reached 0.808 m^2^/g, nearly twice that of OPC. The considerably higher SSA of ISS, together with its smaller median particle size, indicates that ISS possesses a finer particle structure than OPC.

**Figure 2:**
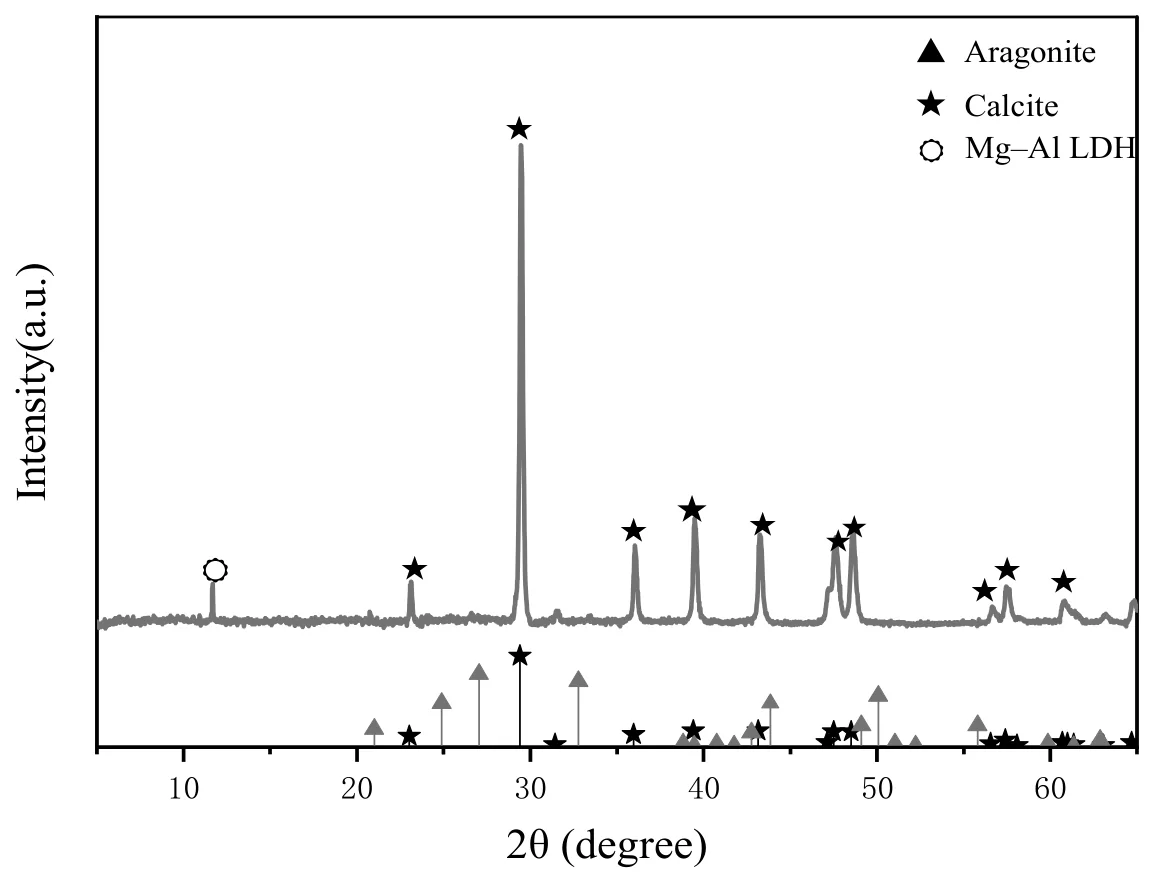
XRD pattern of inorganic softened sludge.

Figure 2 presents the XRD (Rigaku Corporation, Tokyo, Japan) pattern of the raw ISS. Most of the characteristic diffraction peaks can be assigned to calcite, indicating that CaCO_3_ is the predominant crystalline phase in ISS. A weak diffraction peak in the low-angle region is tentatively assigned to a Mg–Al layered double hydroxide (LDH) phase, with a chemical formula of Mg_4_Al_2_(OH)_12_CO_3_·3H_2_O (PDF#01-089-0460). Its structure consists of positively charged Mg–Al hydroxide layers with interlayer carbonate anions and water molecules.

Considering the lime–soda ash softening process and the presence of Mg and Al detected by XRF, this phase may have formed during water treatment. The alkaline environment created by lime addition may promote the hydrolysis and co-precipitation of Mg- and Al-containing species, leading to the formation of positively charged mixed-metal hydroxide layers. Meanwhile, carbonate ions supplied by soda ash may be incorporated into the interlayer regions to compensate for the positive layer charge. Subsequent aging of the wet precipitates may further promote the formation and development of the layered structure [15,16]. In addition, during the water softening process, Mg in ISS may form Mg-containing precipitates, while Si may be incorporated into the sludge through adsorption or co-precipitation. Previous studies have reported Mg(OH)_2_ precipitation and silica removal during water softening processes [17].

**Figure 3:**
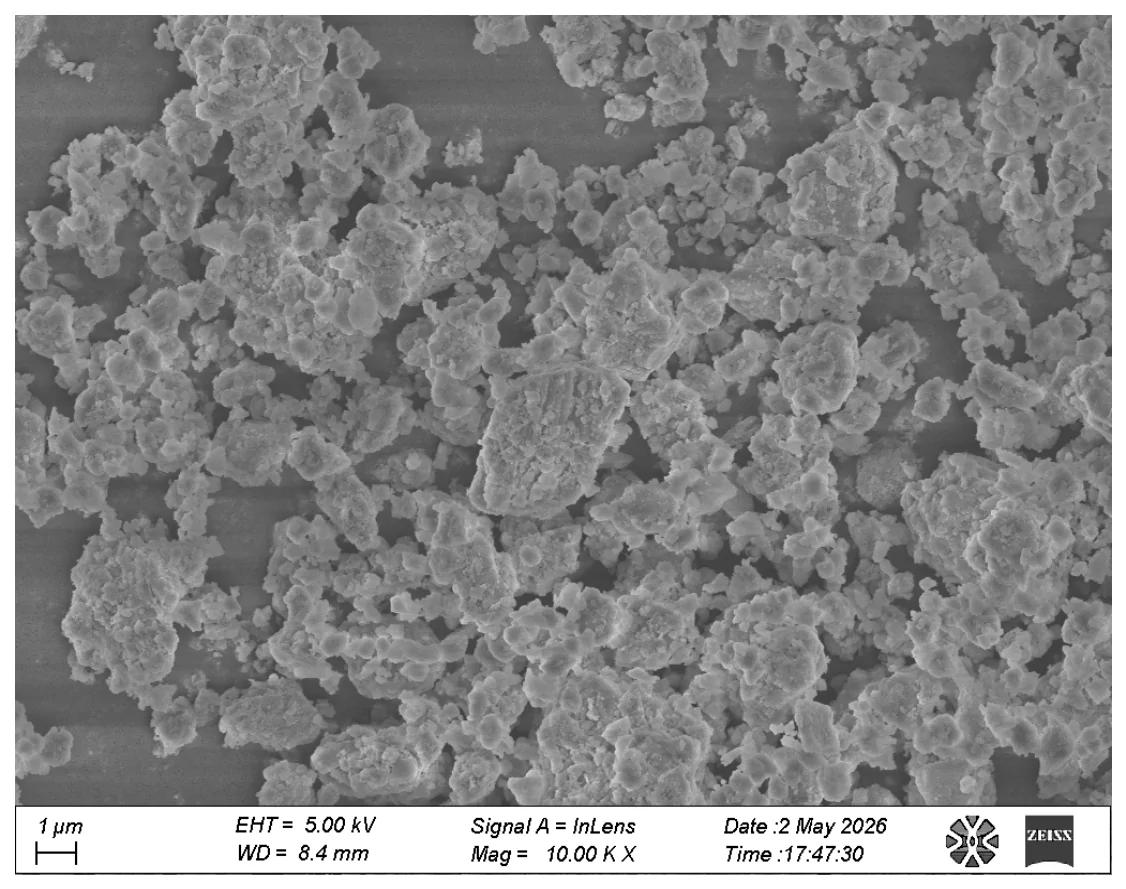
SEM image of inorganic softened sludge.

Figure 3 shows the SEM (Carl Zeiss, Jena, Germany) image of ISS. The ISS particles predominantly occur as irregular agglomerates, with larger agglomerates formed by the accumulation of numerous fine particles. Distinct interparticle boundaries and pore structures can be observed within the agglomerates. In addition, the surfaces of the ISS particles are relatively rough and contain numerous irregular depressions and micropores, exhibiting a complex particle morphology.

### 2.2. Mix Proportion Design and Sample Preparation

Cement paste specimens were prepared according to the mix proportions listed in Table 2, with neat ordinary Portland cement (OPC) paste serving as the reference. Considering that calcite was the main identifiable crystalline phase in ISS, a water-to-binder ratio of 0.40 was adopted with reference to previous studies on cement pastes containing calcium carbonate or limestone particles [18], where the binder mass was defined as the combined mass of OPC and ISS. This maintained a constant water-to-total-powder mass ratio across all mixtures, allowing comparison of fresh properties, strength development, and hydration characteristics at different ISS replacement levels. Prior to sample preparation, the as-received sludge was oven-dried at 110 °C to a constant mass, followed by mechanical grinding and sieving through a 200-mesh standard sieve. During preparation, the required amounts of water, cement, and pretreated ISS were accurately weighed according to the specified mix proportions. The mixtures were then homogenized using a planetary mixer, following the mixing procedure specified in the Chinese standard GB/T 17671-2021 [19] to ensure adequate homogeneity of the cement pastes. After mixing, the fresh cement pastes were cast into 40 mm × 40 mm × 40 mm molds and compacted on a vibrating table to remove entrapped air. The time interval from water addition to completion of molding was strictly controlled to not exceed 10 min. The specimens were cured in a standard curing chamber for 24 h at 20 ± 2 °C and a relative humidity of ≥95%, followed by demolding and further curing under the same conditions until the designated testing ages.

After demolding, the cement paste specimens were transferred to a standard curing chamber and cured at 20 ± 2 °C and a relative humidity of ≥95%. At curing ages of 3 days, 7 days, and 28 days, three specimens were selected for compressive strength testing. Scanning electron microscopy (SEM, Carl Zeiss, Jena, Germany) and X-ray diffraction (XRD, Rigaku Corporation, Tokyo, Japan) were employed to characterize the microstructural morphology and phase evolution of the specimens.

### 2.3. Test Methods

#### 2.3.1. Fluidity

The flowability of the fresh cement pastes was determined in accordance with the Chinese standard GB/T 8077-2012 [20]. After the mold was filled with the freshly mixed paste and the surface was leveled, the mold was lifted vertically at a constant rate, allowing the paste to spread freely on a horizontal glass plate. After 30 s, the spread diameter was measured in two mutually perpendicular directions, and the average of the two measurements was taken as the flow diameter of the paste. Three replicate tests were conducted for each mix proportion, and the results are reported as the mean ± standard deviation.

#### 2.3.2. Setting Time

The setting time was determined in accordance with the Chinese standard GB/T 1346-2011 [21]. The initial and final setting times of the cement-based pastes with different mix proportions were measured using a Vicat apparatus (Wuxi Jianyi Instrument & Machinery Co., Ltd., Wuxi, China). Two Vicat apparatuses were used to perform parallel measurements for each mix proportion, and the final setting time was calculated as the average of the two measurements. If the difference between the two measurements exceeded 15 min, the test was repeated.

#### 2.3.3. Compressive Strength

The compressive strength was determined using cubic specimens with dimensions of 40 mm × 40 mm × 40 mm. At the designated curing ages, the specimens were removed from the standard curing chamber, and excess surface water was wiped off before being placed in a compression testing machine. The loading rate was controlled at 2400 ± 200 N/s until failure occurred. The maximum failure load was recorded, and the compressive strength was calculated accordingly. Three specimens were tested for each mix proportion at curing ages of 3 days, 7 days, and 28 days, and the results are reported as the mean ± standard deviation.

#### 2.3.4. XRD

X-ray diffraction (XRD) analysis was performed using a SmartLab SE X-ray diffractometer (Rigaku Corporation, Tokyo, Japan). The measurements were conducted at a tube voltage of 40 kV and a tube current of 200 mA using Cu Kα radiation. The scanning rate was set to 1 °/min, with a 2θ range of 5–65°.

#### 2.3.5. FTIR

Fourier transform infrared (FTIR) spectroscopy was performed using a Nicolet iS50 spectrometer (Thermo Fisher Scientific, Waltham, MA, USA). The samples were prepared using the KBr pellet method by uniformly mixing 1 mg of powdered sample with 100 mg of KBr, followed by pellet pressing. The spectra were recorded over the wavenumber range of 4000–400 cm^−1^ at a spectral resolution of ≤0.09 cm^−1^, with 32 scans accumulated for each measurement.

#### 2.3.6. TG-DTG

Thermogravimetric analysis coupled with differential thermogravimetry (TG-DTG) was performed using an STA 449 F3 simultaneous thermal analyzer (Netzsch Instrument GmbH, Selb, Germany). The measurements were conducted over a temperature range of 30–1000 °C at a heating rate of 10 °C/min. Argon was used as the purge gas at a flow rate of 50 mL/min, while nitrogen was used as the protective gas at a flow rate of 20 mL/min.

#### 2.3.7. SEM-EDS/BSE

Scanning electron microscopy coupled with energy-dispersive X-ray spectroscopy (SEM-EDS) and backscattered electron (BSE) imaging were performed using a SIGMA 500 field-emission scanning electron microscope (Carl Zeiss, Jena, Germany). The surface morphology of the samples was examined by SEM, while energy-dispersive X-ray spectroscopy (EDS) was used to determine the elemental composition of selected regions.

## 3. Results

### 3.1. Fluidity

**Figure 4:**
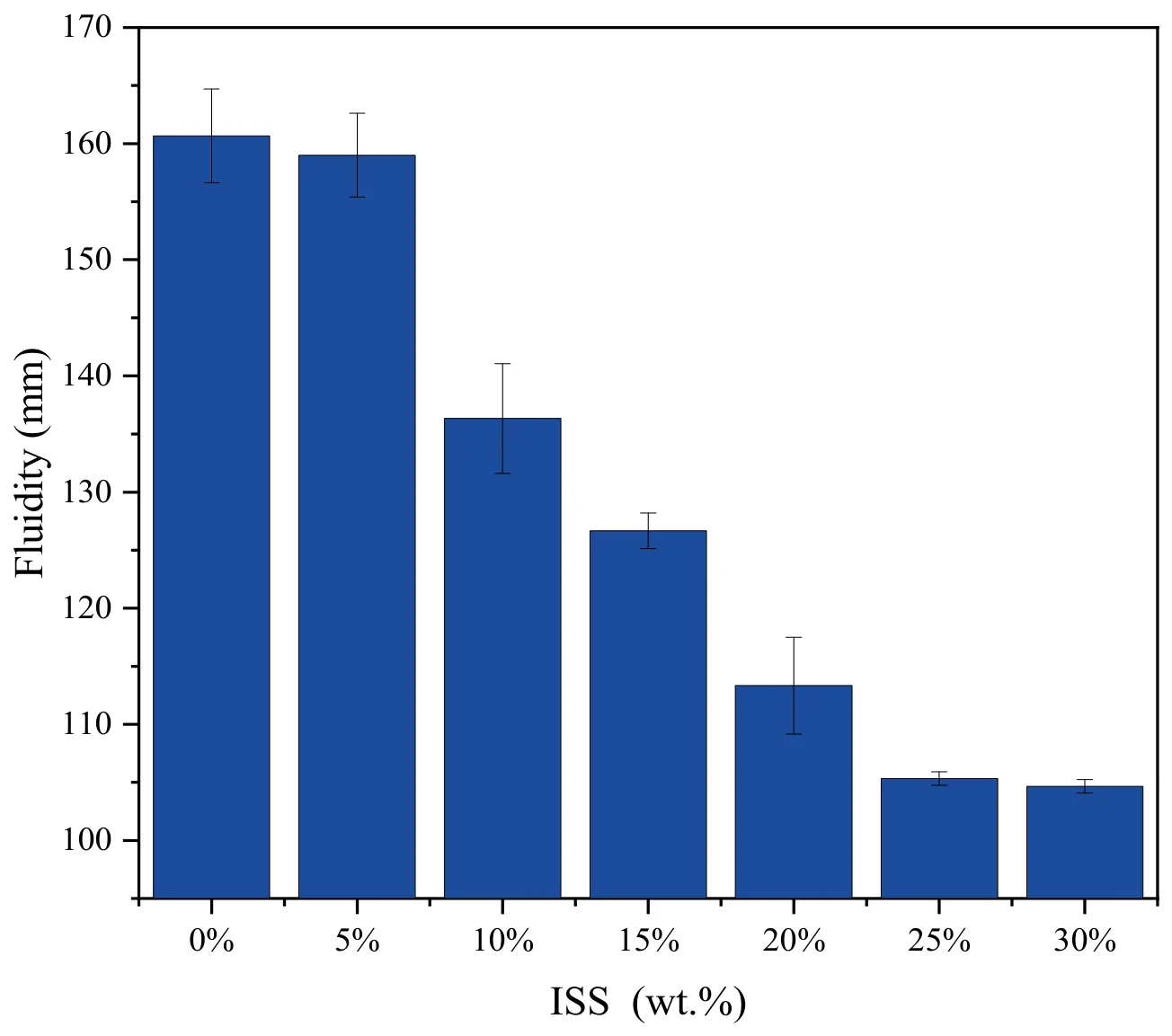
Effect of inorganic softened sludge content on flowability.

As shown in Figure 4, the incorporation of ISS reduced the initial flowability of the cement paste. In the absence of ISS, the reference paste exhibited a flow diameter of 160 mm. As the ISS content increased, the flow diameter gradually decreased to 136, 113, and 104 mm at ISS contents of 10%, 20%, and 30%, respectively, corresponding to reductions of approximately 15%, 29%, and 35% compared with the reference paste. The flowability of the paste exhibited a continuous decreasing trend. Combined with the SEM observations of the microstructure, the reduction in flowability can be primarily attributed to the irregular morphology and porous, rough surface characteristics of the ISS particles. The flocculation-derived agglomerates hinder interparticle sliding and increase the internal structural resistance of the paste, thereby resulting in reduced flowability of the fresh paste [22].

On the other hand, under equal-mass replacement conditions, the incorporation of ISS substantially increases the specific surface area of the particles in the composite paste, resulting in a greater proportion of water being consumed for particle-surface wetting and adsorption. According to the water-film theory, when the water-to-binder ratio is kept constant, a higher specific surface area reduces the proportion of free water available for lubrication between particles, thereby increasing interparticle interactions and consequently decreasing the flowability of the system [23,24].

### 3.2. Setting Time

**Figure 5:**
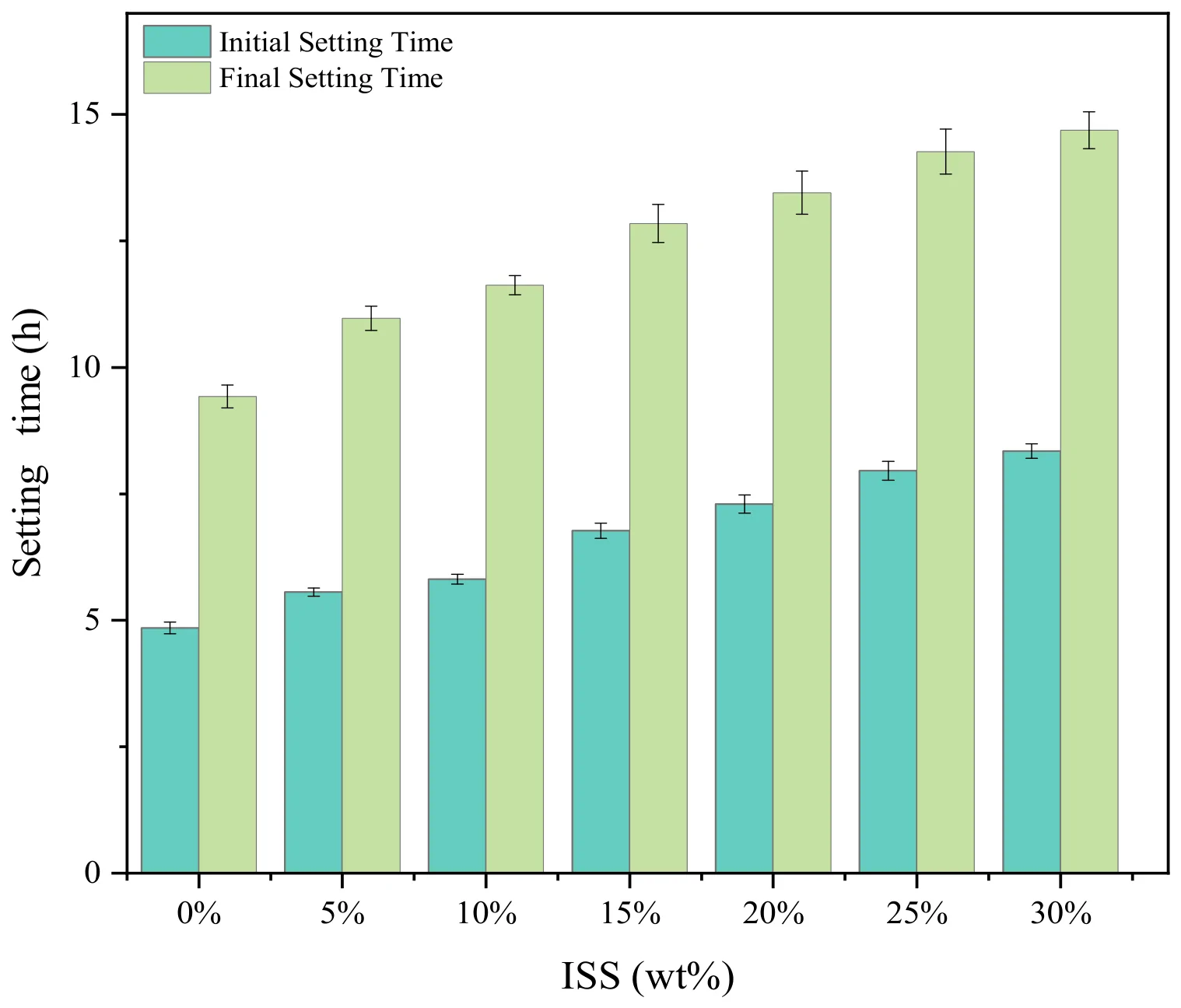
Effect of ISS content on the setting time of the cement paste.

Setting time is a key indicator reflecting the rate of early-age hydration reactions of cementitious materials [25,26]. Figure 5 illustrates the evolution of the setting time of the OPC-ISS composite pastes. For the reference OPC paste, the initial and final setting times were 4.8 hand 9.5 h, respectively. The incorporation of ISS prolonged both the initial and final setting times, with a monotonic increase observed as the ISS replacement level increased. Compared with the reference OPC paste, the paste containing 30% ISS exhibited substantial increases in both the initial and final setting times, reaching 8.4 h and 14.7 h, respectively. These values correspond to increases of approximately 75.0% and 54.7%, respectively, relative to the OPC control. This phenomenon may be attributed to the reduction in clinker content caused by the replacement of OPC with ISS, as well as the relatively high specific surface area of ISS, which increases the overall specific surface area of the composite paste. Previous studies have shown that fine-particle fillers, owing to their relatively large specific surface area, can alter the effective distribution of water within the paste and consequently affect the hydration kinetics of cementitious systems [27]. Therefore, the particle characteristics of ISS may exert a retarding effect on the setting process of the cement paste.

### 3.3. Compressive Strength

**Figure 6:**
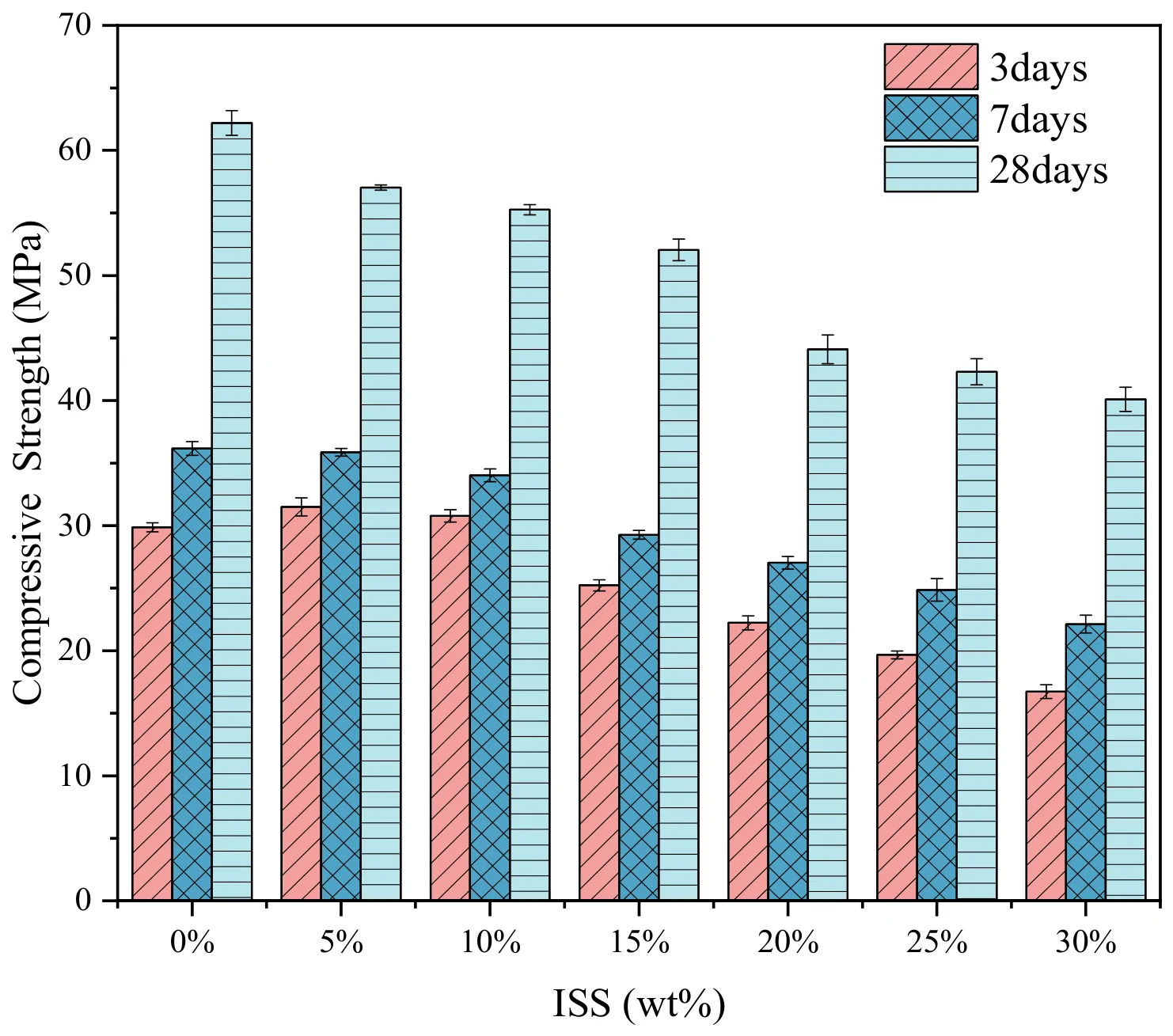
Compressive strength of the composite pastes.

Figure 6 presents the evolution of the compressive strength of OPC-ISS composite pastes at curing ages of 3 days, 7 days, and 28 days. As expected, the compressive strength of all cementitious specimens steadily increased with increasing curing age. At 3 days, the compressive strength initially increased and then decreased with increasing ISS content, indicating the existence of an optimum ISS replacement level for early-age strength development. The 3 days compressive strength of the paste containing 5% ISS reached 31.5 MPa, which was higher than that of the OPC reference paste (29.8 MPa). Meanwhile, the composite paste containing 5% ISS exhibited comparable later-age compressive strength to the OPC paste, indicating that the incorporation of a small amount of ISS had a negligible effect on the overall strength development.

The slight increase in early-age compressive strength at low ISS replacement levels may be partly associated with the surface effects of calcite particles. Previous studies have shown that calcite surfaces can serve as substrates for C-S-H nucleation and growth [28]. As calcite is the predominant crystalline phase in ISS, ISS particles may provide additional substrates for C-S-H formation and growth, potentially contributing to early-age strength development at low replacement levels. At the later curing age, the compressive strengths of the specimens containing 5–10% ISS showed only a slight reduction and remained comparable to that of the OPC reference paste, indicating that a low ISS content had a limited influence on later-age strength development. However, at higher ISS replacement levels, the 28 days compressive strength of the composite pastes exhibited a substantial loss. This can be attributed to the excessive replacement of cement by ISS, which reduces the clinker mineral content and consequently limits the formation of hydration products required to maintain a dense cementitious structure [29] thereby leading to a reduction in later-age compressive strength.

### 3.4. XRD

**Figure 7:**
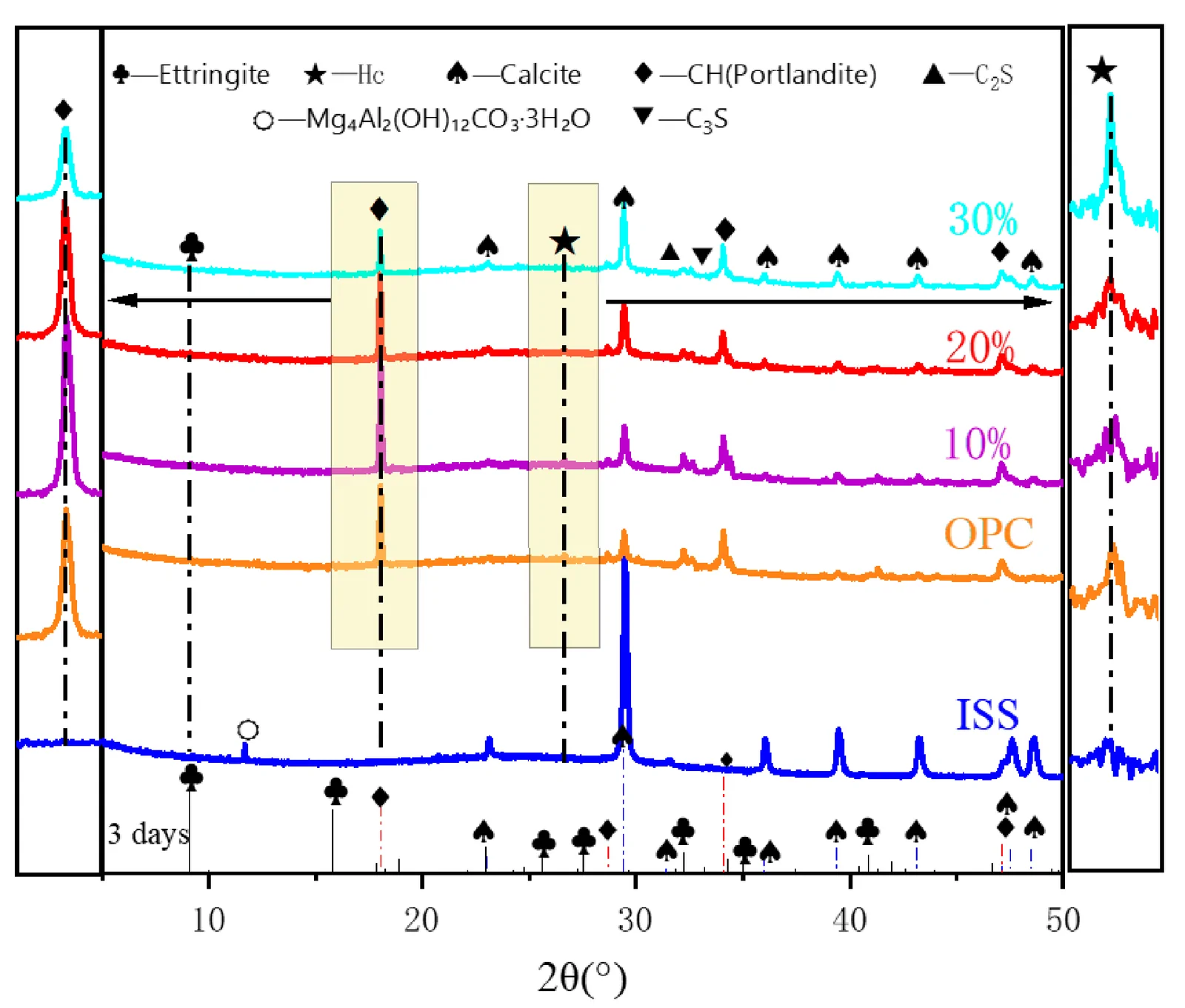
XRD patterns of the composite pastes at 3 days.

**Figure 8:**
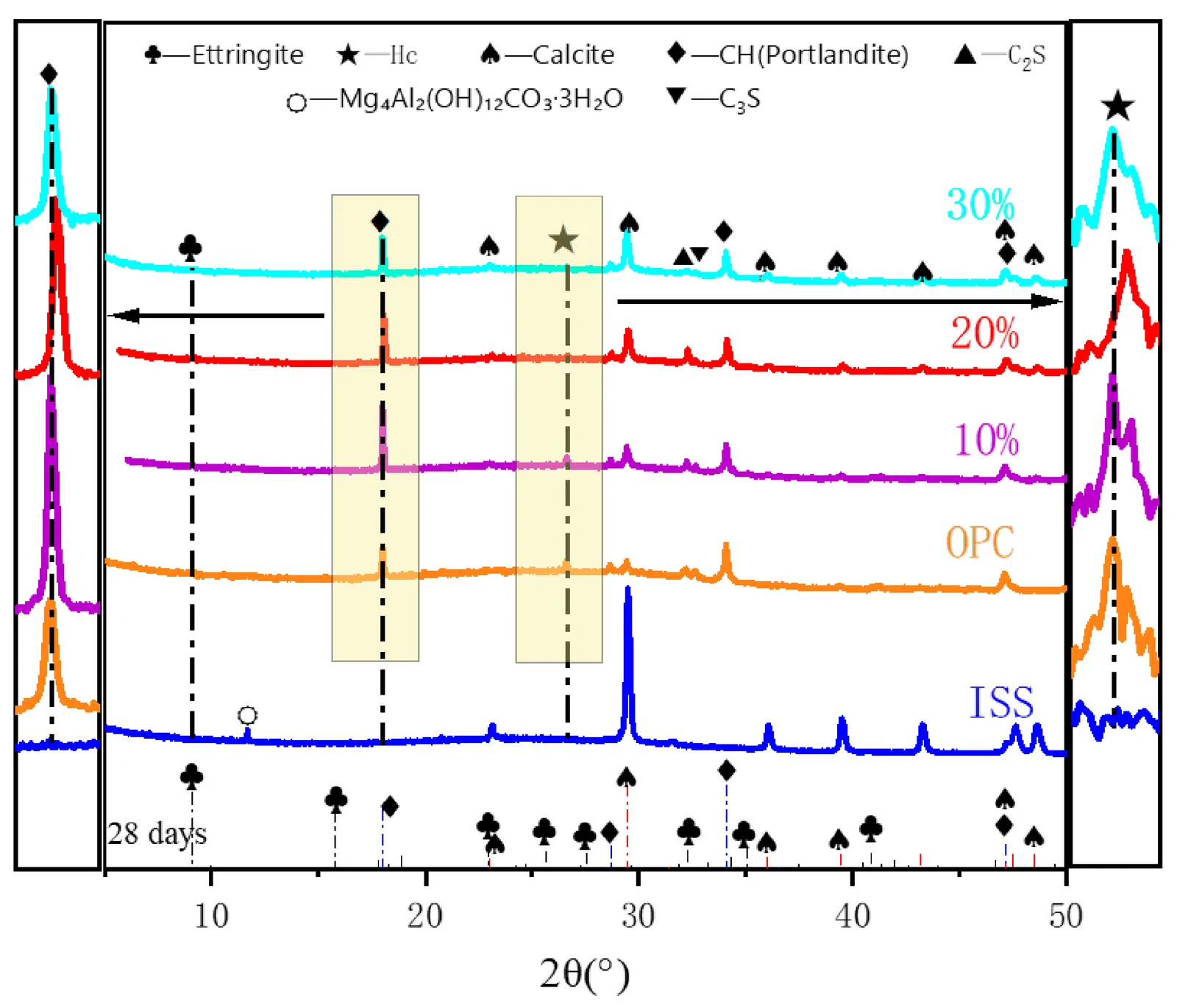
XRD patterns of the composite pastes at 28 days.

The XRD patterns of the hydration products in the OPC-ISS composite pastes at 3 days and 28 days are presented in Figure 7 and Figure 8, respectively, together with those of the OPC reference paste and the raw ISS sample for comparison. All samples exhibited characteristic diffraction peaks corresponding to portlandite (CH, PDF#44-1481) [30], ettringite (AFt, PDF#41-1451) [30], as well as unhydrated clinker phases, including C_2_S and C_3_S, In addition, a weak diffraction peak associated with a carbonate-bearing Mg–Al layered double hydroxide (LDH) phase was detected in the raw ISS, with a chemical formula of Mg_4_Al_2_(OH)_12_CO_3_·3H_2_O (PDF#01-089-0460). Following the incorporation of ISS into the cement system, characteristic diffraction peaks of calcite were detected in all ISS-containing samples, with their peak intensities progressively increasing with increasing ISS content. In addition, diffraction peaks associated with hemicarboaluminate (Hc), a carbonate-bearing AFm-type hydrate, were observed in the ISS-containing pastes. Its formation may be associated with the participation of carbonate species released through the partial dissolution of calcite in ISS in the hydration reactions of cement aluminate phases (C_3_A) under calcium-rich alkaline conditions [31]. This suggests that the carbonate components introduced by ISS can participate in the evolution of aluminate-bearing hydration products.

At 3 days, the intensity of the CH diffraction peak initially increased and then decreased with increasing ISS replacement, reaching a maximum at 10% replacement. Although this mixture contained less cement than the control, its CH diffraction peak did not decrease accordingly, suggesting that ISS incorporation at a low replacement level may influence the formation of early-age hydration products. At higher ISS replacement levels, the initial amount of silicate clinker phases decreased progressively, accompanied by a reduction in CH peak intensity. At 28 days, the characteristic diffraction peaks of hemicarboaluminate (Hc) remained observable in the ISS-containing pastes, with only slight variations in peak intensity as the ISS replacement level increased. The persistent detection of Hc indicates that carbonate-bearing AFm-type hydrates were still present at this curing age. Their formation may be associated with the participation of carbonate species released through the partial dissolution of calcite in the evolution of aluminate-bearing hydration products [32]. For the pure OPC paste, the intensity of the AFt characteristic peak decreased at 28 days compared with that at 3 days. Meanwhile, the diffraction intensities of the unhydrated clinker phases, C_3_S and C_2_S, further decreased, indicating that the residual clinker minerals continued to participate in hydration as the curing age increased.

### 3.5. FTIR

**Figure 9:**
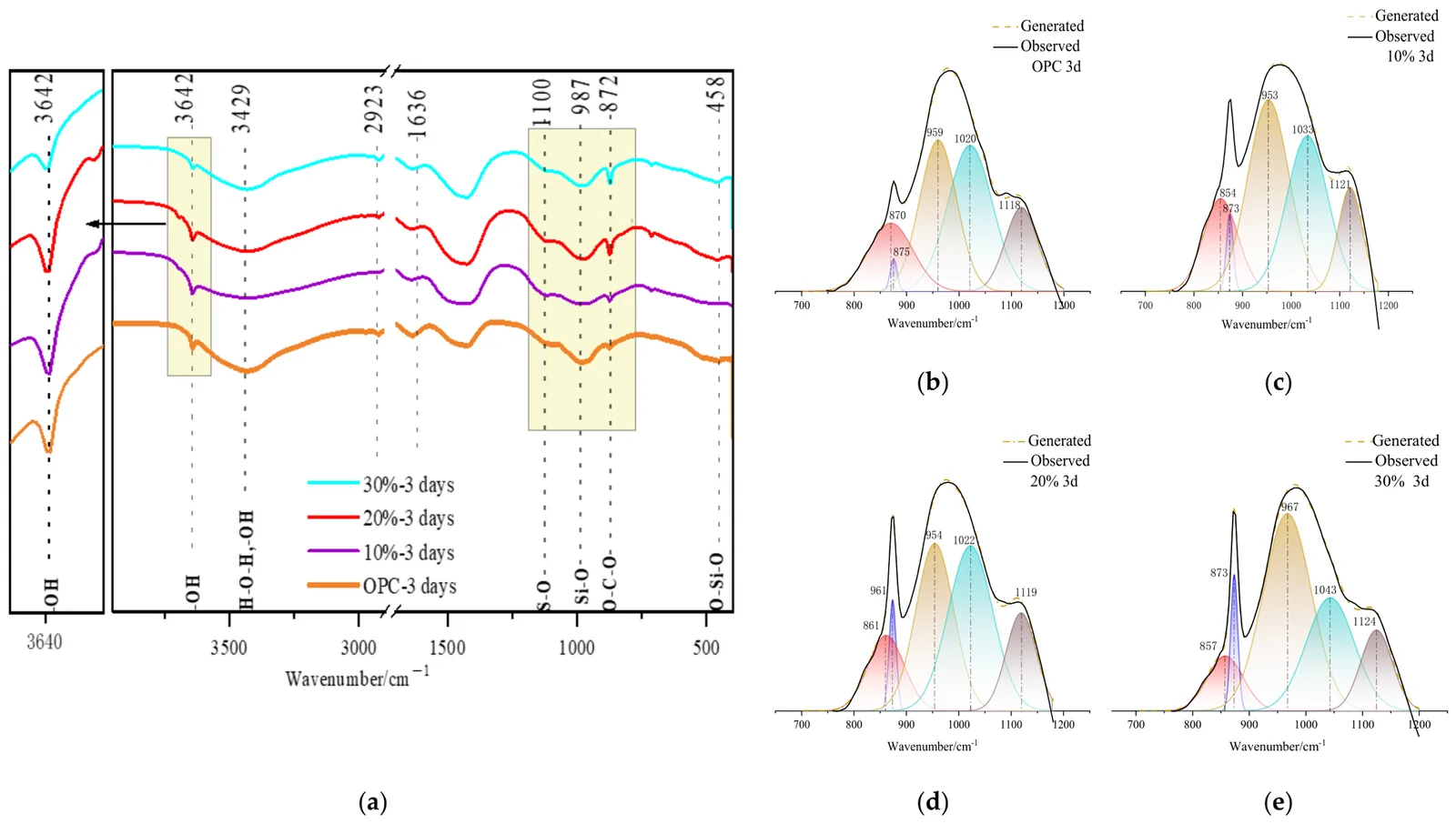
FTIR spectra of the composite pastes at 3 days.

**Figure 10:**
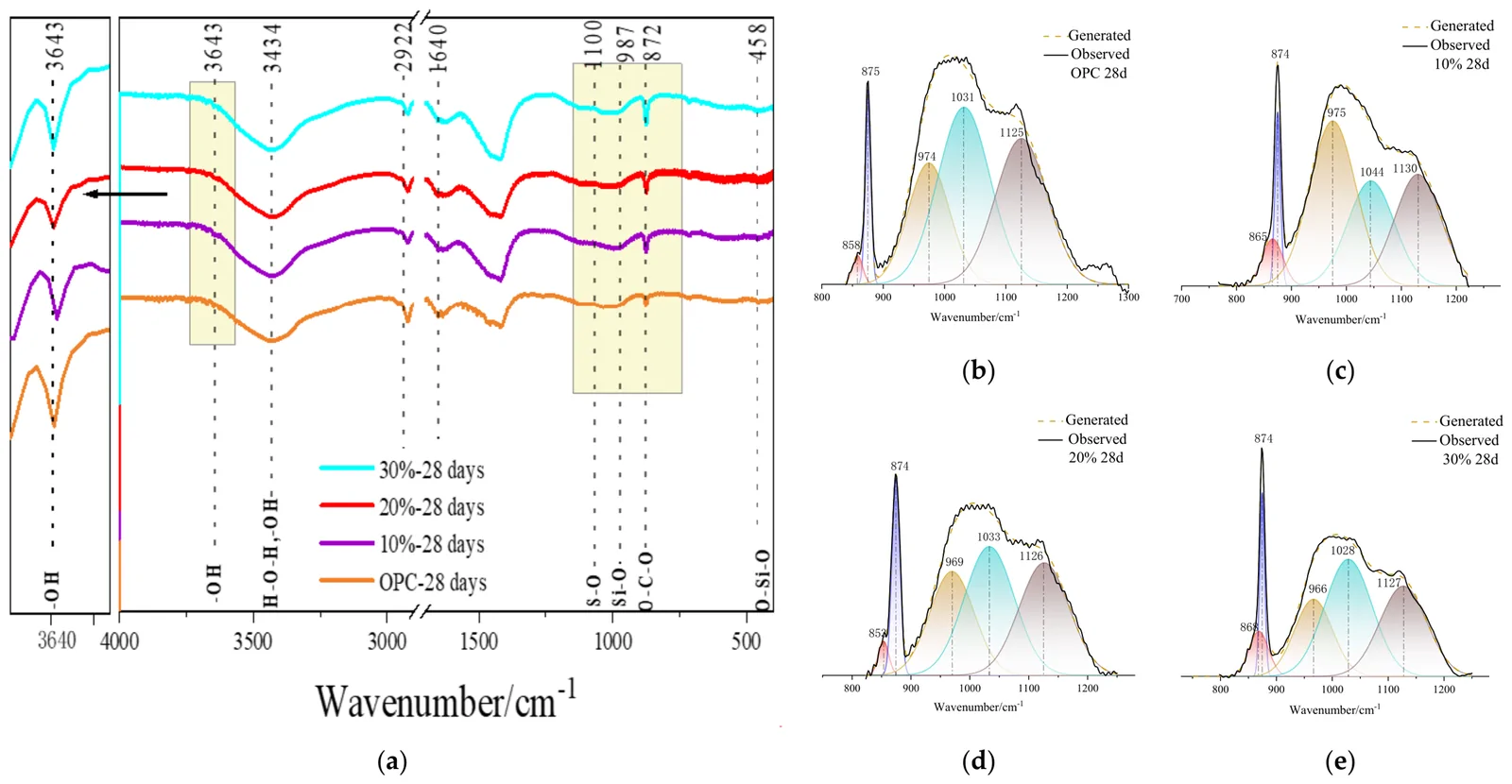
FTIR spectra of the composite pastes at 28 days.

Figure 9 and Figure 10 show the FTIR spectra of the composite pastes at curing ages of 3 days and 28 days, respectively, with the insets on the left providing enlarged views of the absorption band at approximately 3640 cm^−1^. The major absorption bands were assigned as follows. The sharp absorption band at approximately 3640 cm^−1^ is attributed to the O-H stretching vibration of structural hydroxyl groups in calcium hydroxide (CH) [33]. The enlarged spectra reveal that a distinct narrow absorption band at approximately 3642–3643 cm^−1^ can be identified in all samples at both curing ages, consistent with the detection of CH in the XRD analysis. The absorption bands located at approximately 1650 cm^−1^ and 3446 cm^−1^ correspond to the bending vibration of H-O-H in molecular water and the stretching vibration of O-H groups, respectively [33]. All samples exhibited distinct absorption bands in the ranges of 1600–1700 cm^−1^ and 3000–3500 cm^−1^, which are mainly associated with bound water and adsorbed water in the hydration products [33]. The absorption band near 872 cm^−1^ is attributed to the out-of-plane bending vibration of O–C–O bonds in CO_3_^2−^ groups, The absorption band near 1120 cm^−1^ corresponds to the S–O stretching vibration of SO_4_^2−^ groups. The absorption band at 987 cm^−1^ is assigned to the asymmetric stretching vibration of Si-O-T (T = Si or Al) bonds [33].

In the range of 800–1100 cm^−1^, all systems exhibited a characteristic absorption band associated with the asymmetric stretching vibration of Si-O bonds in C-S-H gels [34]. The incorporation of ISS caused no noticeable shift in the position of this characteristic band, indicating that the introduction of ISS did not substantially alter the local structural environment of the silicate tetrahedra in the C-S-H gel. However, at 28 days, the intensity of the absorption band in this region gradually decreased with increasing ISS replacement levels. Considering the aforementioned greater loss in 28 days compressive strength at higher ISS replacement levels, this change may be associated with the reduced initial clinker content and the limited formation of silicate hydration products. Nevertheless, the specific changes in silicate structural units cannot be readily distinguished from the original FTIR spectra alone. Therefore, peak deconvolution was further performed on the relevant overlapping absorption bands to semi-quantitatively compare the relative contributions of the bands associated with Q^0^, Q^1^, and Q^2^ structural units.

To further investigate the influence of ISS incorporation on the hydration process of the cementitious system, peak deconvolution was performed in the FTIR spectral region of 800–1200 cm^−1^ to semi-quantitatively analyze the distribution of Q^n^ structural units with different degrees of polymerization by resolving overlapping absorption bands. The fitting procedure was conducted using the Peak Analyzer function in Origin software. First, two baseline anchor points were manually selected, and a linear baseline was established by interpolation and subsequently subtracted. Unweighted multi-peak fitting was then performed using Gaussian peak functions and the Levenberg–Marquardt algorithm. The upper limit of the full width at half maximum (FWHM) for each component peak was set to 100 cm^−1^, with a maximum of 20,000 iterations and a convergence tolerance of 10^−6^. The peak deconvolution results are presented in Figure 9 and Figure 10, providing a basis for evaluating the structural evolution of hydration products following ISS incorporation.

**Figure 11:**
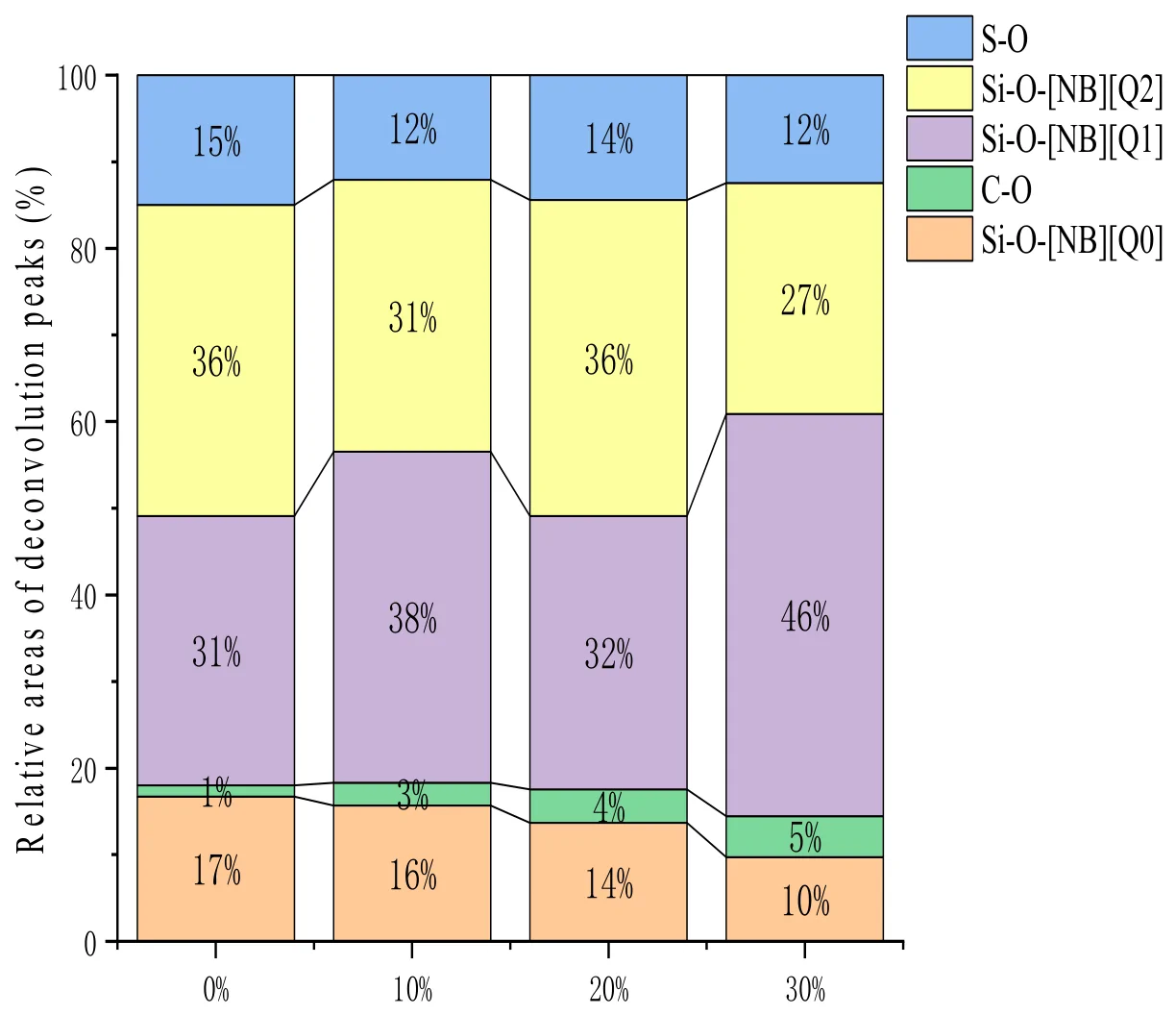
Relative distribution of deconvoluted FTIR spectral components of the composite pastes at 3 days.

**Figure 12:**
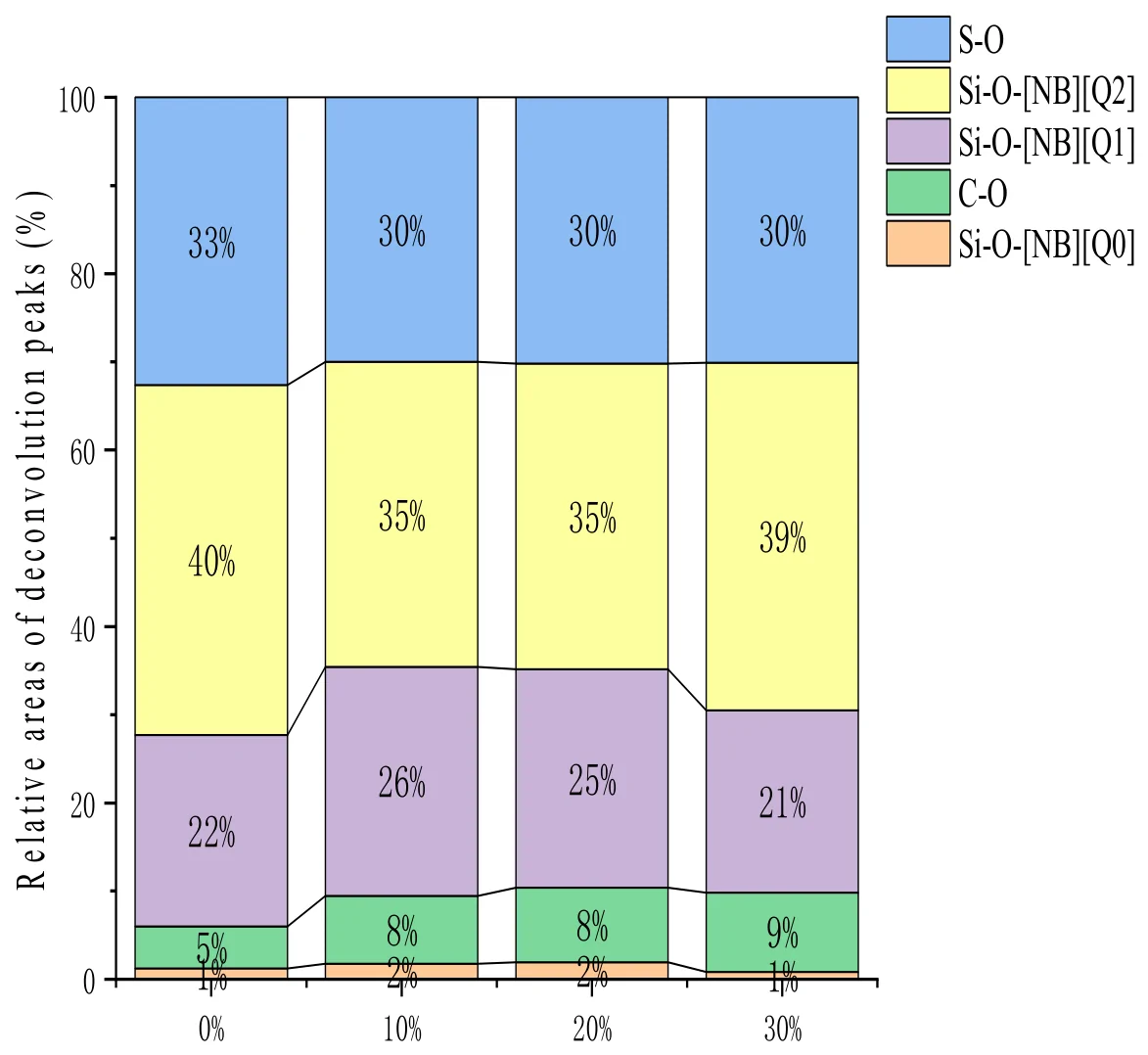
Relative distribution of deconvoluted FTIR spectral components of the composite pastes at 28 days.

Figure 11 and Figure 12 show the peak deconvolution results of the main silicate absorption band (800–1200 cm^−1^) for the composite pastes at 3 days and 28 days, respectively. This analysis enables a quantitative characterization of the relative distribution of the silicate network structures and hydration products in the cementitious system. The assignments of the individual deconvoluted bands were determined based on previous studies:

840–870 cm^−1^: assigned to the Si-O vibrations of unhydrated silicate clinker phases, corresponding to isolated Q^0^ tetrahedra [34];

868–876 cm^−1^: assigned to the out-of-plane bending vibration of C-O bonds in CO_3_^2−^ groups derived from ISS [35];

900–940 cm^−1^: assigned to the Si-O stretching vibrations of primary C-S-H structures, corresponding to Q^1^ dimers or short silicate chains [34];

950–990 cm^−1^: assigned to the Si-O stretching vibrations of the mature C-S-H network, corresponding to polymerized Q^2^ long silicate chains [36];

1100–1150 cm^−1^: assigned to the asymmetric stretching vibration of S-O bonds in SO_4_^2−^ groups within the AFt structure [37].

At 3 days, in the neat OPC paste, the Q^2^ units representing the more developed silicate network accounted for 36%, exceeding the proportion of Q^1^ units (31%). This indicates that the silicate tetrahedra underwent progressive polymerization during hydration, accompanied by the development of the C-S-H gel structure and an increase in the degree of silicate chain polymerization [38]. With increasing ISS content, the proportion of Q^1^ units increased to 46% at an ISS content of 30%, whereas the proportion of Q^2^ units decreased to 27%. This indicates that the C-S-H gel formed in the high-ISS system was predominantly characterized by short-chain structures. The increased ISS content therefore inhibited the further polymerization of C-S-H, thereby suppressing the extension of silicate chains.

At 28 days, the silicate structures in all systems underwent further evolution. Compared with the 3 days results, the relative proportion of the Q^0^ units decreased markedly from 10–17% to 1–2%, indicating that the remaining unhydrated silicate phases continued to participate in hydration. Meanwhile, the progressive formation of C-S-H gel promoted further polymerization of the silicate tetrahedra within the system. Although increasing ISS content reduced the clinker content of the composite system, ISS did not alter the fundamental hydration pathway of cement; its primary effect was manifested as a reduction in the degree of silicate polymerization due to the dilution of clinker [39]. Notably, the relative area of the C-O characteristic band associated with the carbonate phase gradually increased with increasing ISS content. Its relative area increased from 5% in the reference OPC paste to 9% at an ISS content of 30%, this is consistent with the increased amount of carbonate introduced into the paste. Combined with the XRD results, this observation indicates that calcite remained detectable after hydration and that carbonate species may participate in the evolution of aluminate-bearing hydration products. In addition, compared with 3 days, the relative fitted area of the S-O-related bands in all systems increased from 12–15% to 30–33% at 28 days. As hydration progressed, the composition of the silicate-related bands continued to evolve, while the relative contribution of sulfate-related vibrations within the analyzed spectral region increased. Together with the persistent AFt characteristic diffraction peaks observed in the XRD patterns at 28 days, the FTIR results further support the retention of sulfate-bearing hydration products at later ages. For ISS-containing pastes, previous studies have reported that the participation of carbonate species in the formation of carboaluminate phases can favor the stability of AFt [40]. In the present study, the coexistence of hemicarboaluminate (Hc) and AFt is consistent with these findings, suggesting that the carbonate components introduced by ISS may contribute to the retention of AFt during later-stage hydration.

### 3.6. TG-DTG

**Figure 13:**
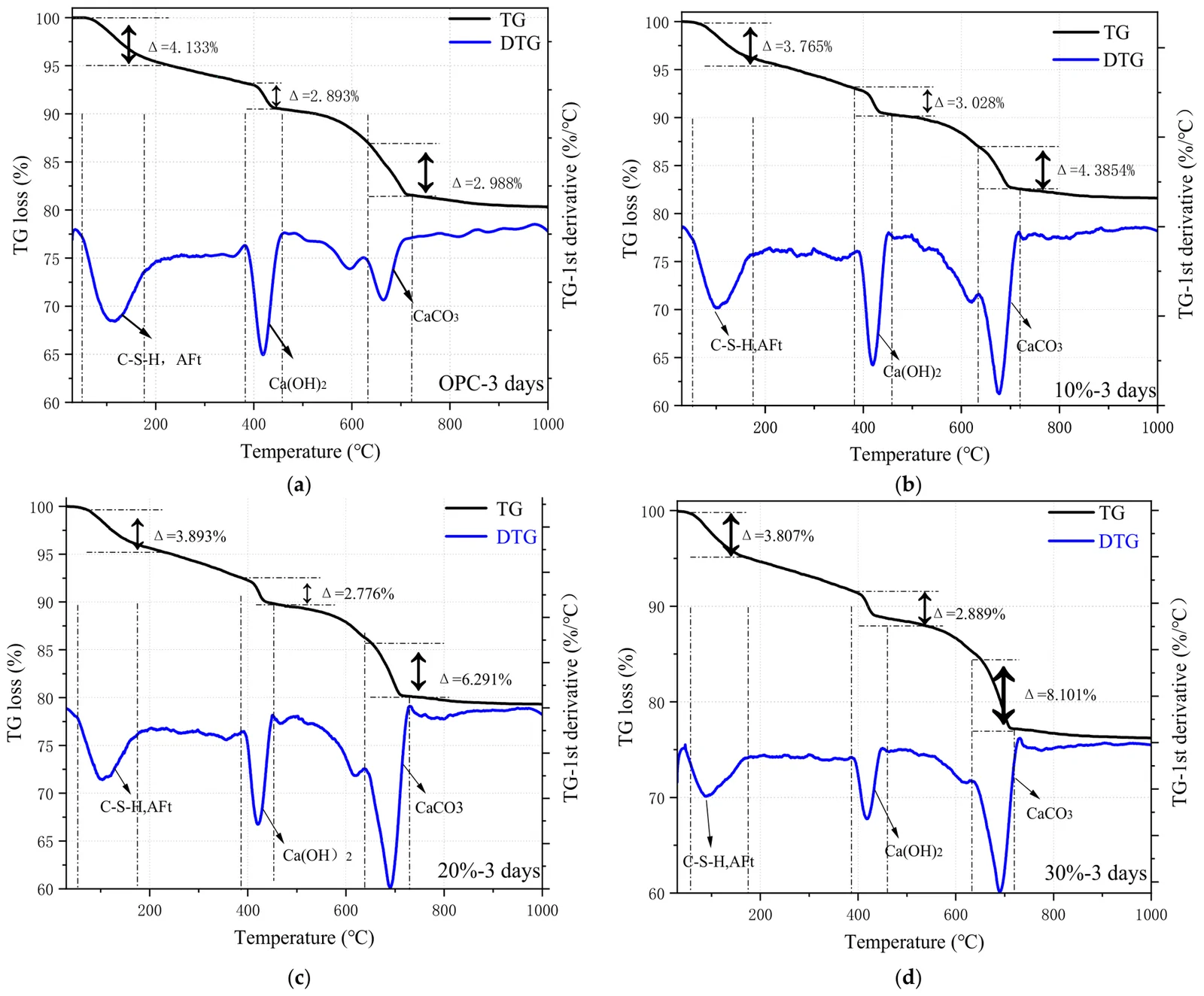
TG-DTG curves of the composite pastes at 3 days.

**Figure 14:**
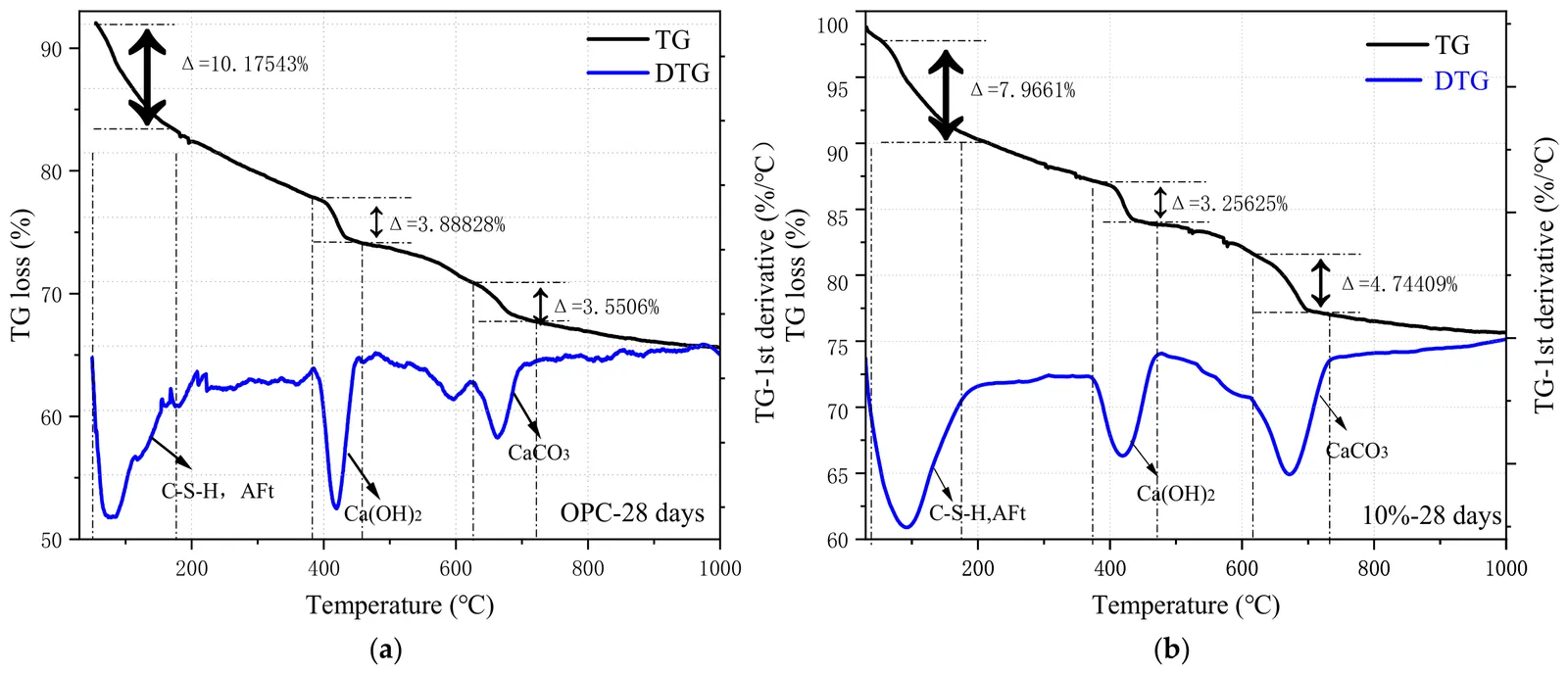

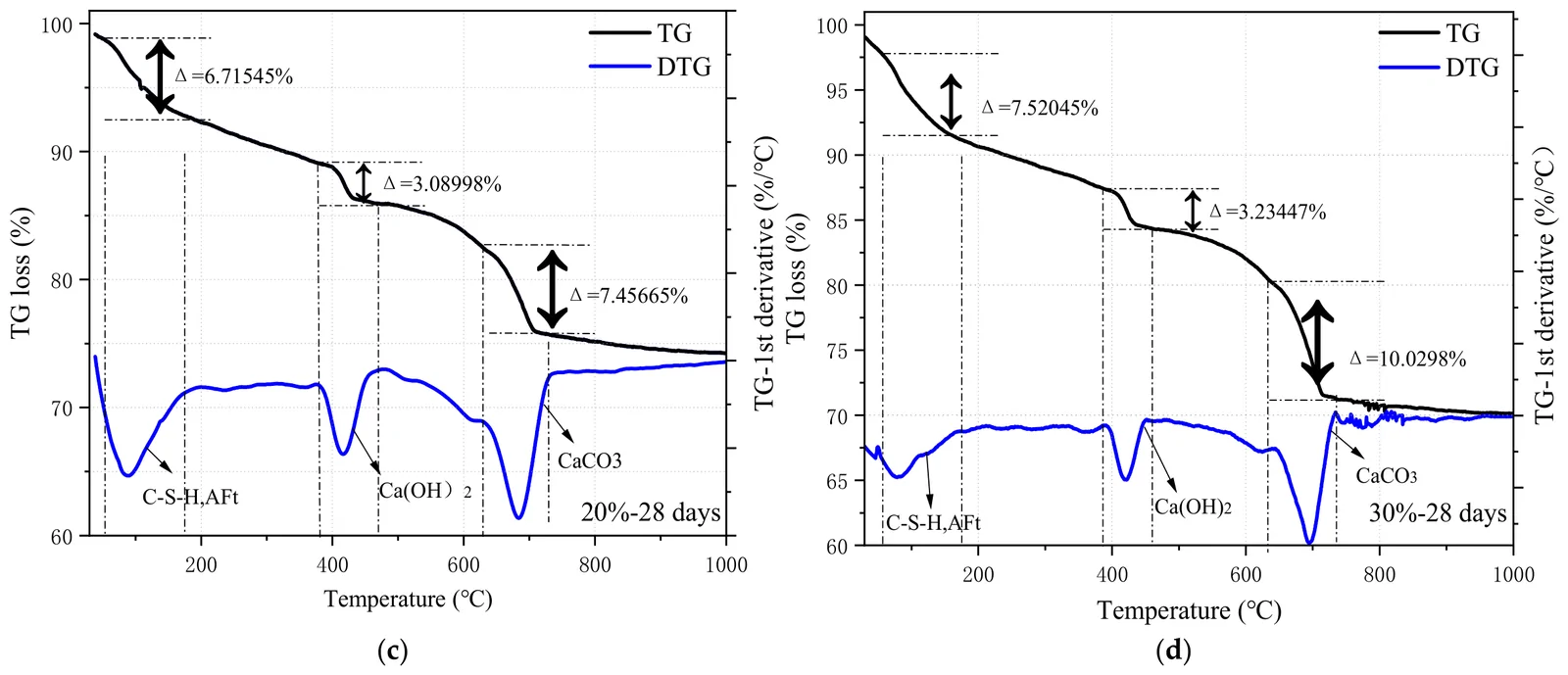
TG-DTG curves of the composite pastes at 28 days.

Figure 13 and Figure 14 show the TG-DTG curves of the OPC-ISS composite pastes at 3 days and 28 days, respectively. All samples exhibited three distinct major thermal decomposition stages, corresponding to the temperature ranges of 50–200 °C (dehydration of C-S-H gel, AFt, and AFm phases), 400–500 °C (dehydroxylation of CH), and 600–800 °C (decarbonation of carbonate phases) [41,42]. These observations indicate that the main mass-loss temperature ranges were similar across the samples, whereas the CH-related mass loss within 400–500 °C and the carbonate-related mass loss within 600–800 °C exhibited different trends with increasing ISS replacement.

At 3 days, as shown in Table 3, the mass losses associated with CH decomposition within 400–500 °C were 2.893%, 3.028%, 2.776%, and 2.889% for the OPC reference and the pastes containing 10%, 20%, and 30% ISS, respectively. The value for the 10% ISS paste was 0.135% higher than that of the OPC reference, consistent with the stronger CH diffraction peak observed by XRD. Despite the lower initial cement content, the slight increase in CH-related mass loss at 10% ISS suggests a change in early-age CH formation or accumulation. At replacement levels of 20% and 30%, the CH-related mass losses were lower than that of the 10% ISS paste, consistent with the further reduction in the initial amount of silicate clinker phases.

At 28 days, as shown in Table 4, all ISS-containing pastes exhibited lower mass losses associated with CH decomposition within 400–500 °C than the OPC reference. The CH-related mass loss was 3.88% for the OPC reference and 3.26%, 3.09%, and 3.23% for the pastes containing 10%, 20%, and 30% ISS, respectively, corresponding to reductions of 16.0%, 20.4%, and 16.8%. In addition, the mass loss within 600–800 °C increased markedly with increasing ISS replacement. At 3 days, it increased from 2.99% for the OPC reference to 8.10% for the 30% ISS paste; at 28 days, the corresponding values increased from 3.55% to 10.03%. This observation indicates that ISS introduced a substantial amount of stable carbonate minerals, which the increase in carbonate-related mass loss indicates that a substantial fraction of the carbonate introduced by ISS remained detectable after hydration and underwent decarbonation upon heating.

### 3.7. SEM-EDS/BSE

**Figure 15:**
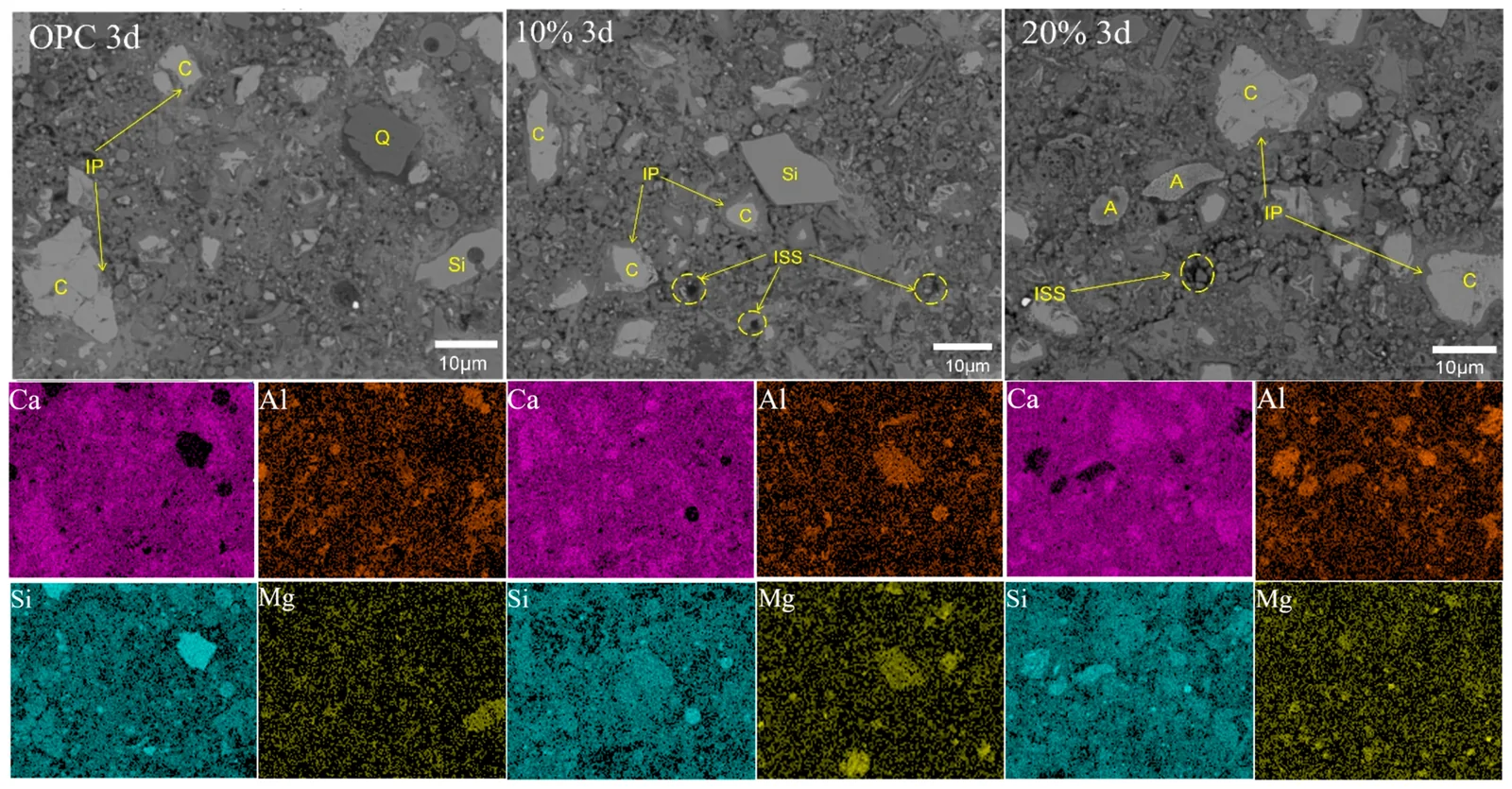
BSE micrographs of the composite pastes at 3 days (C: unhydrated cement clinker; IP: internal products; A: Al-Si-containing phase; Q: quartz; ISS: unreacted sludge particles; Si: unreacted silicate clinker particles).

**Figure 16:**
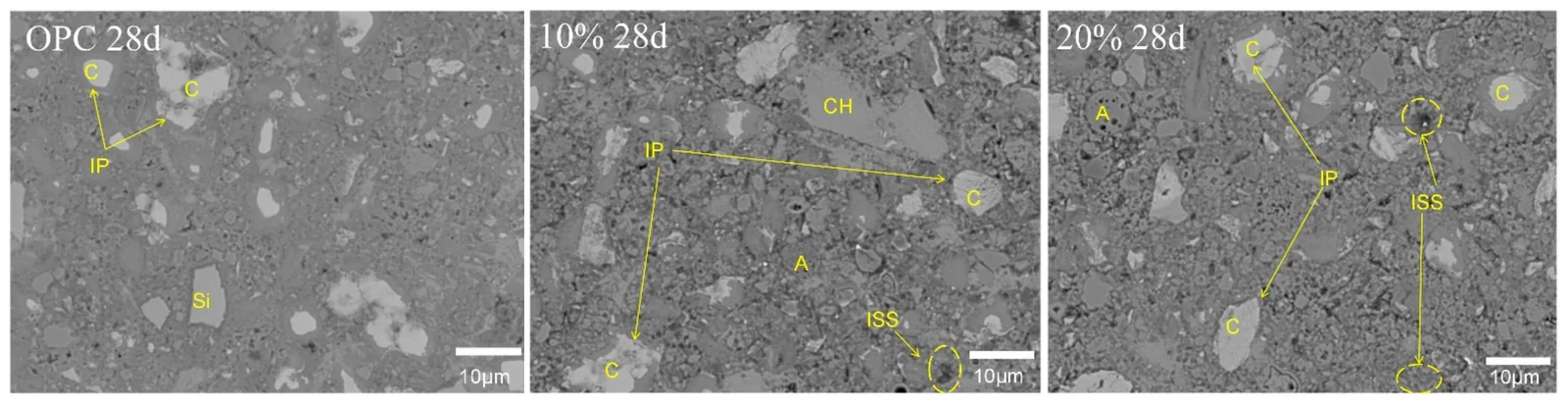

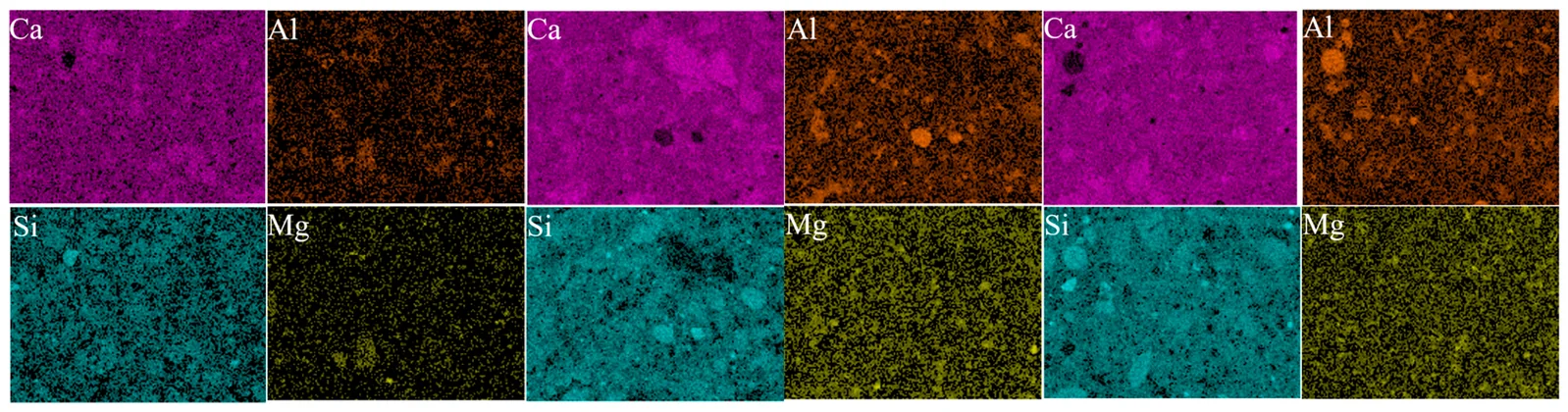
BSE micrographs of the composite pastes at 28 days (C: unhydrated cement clinker; IP: internal products; A: Al-Si-containing phase; Q: quartz; ISS: unreacted sludge particles; Si: unreacted silicate clinker particles).

The BSE micrographs and corresponding EDS elemental maps of the polished OPC-ISS composite pastes are presented in Figure 15 and Figure 16. The different regions exhibit distinct grayscale contrasts owing to differences in their average atomic numbers. The brighter regions are primarily associated with unhydrated clinker particles, whereas the continuous gray matrix mainly consists of hydration products, including C-S-H gel, CH, and a small amount of aluminate-containing hydrates. The darkest regions are predominantly attributed to the ISS particles [43].

For the OPC-3 d sample, a large number of unhydrated clinker particles can be observed dispersed within the continuous hydration matrix, with distinct regions of hydration products formed around the clinker particles. The EDS results show that the matrix regions are primarily composed of Ca, Si, and O, indicating that they mainly consist of calcium silicate hydrate (C-S-H) products. In addition, some Ca-rich regions with relatively low Si contents can be attributed to CH, which is consistent with the results of the XRD and TG-DTG analyses.

In the ISS-containing pastes, irregular agglomerated particle regions with indistinct boundaries were observed in the BSE images. The EDS elemental maps showed that these regions were enriched in Ca and Mg, while exhibiting relatively weak Si signals, suggesting that they were predominantly unreacted ISS particles. Some carbonate particles were uniformly dispersed within the hydrated matrix, with hydration-product regions observed adjacent to them. EDS elemental mapping revealed co-enrichment of Ca and Si in these regions, consistent with the elemental composition of C-S-H-like hydration products. With increasing ISS content, the hydration-product matrix appeared less continuous, while regions associated with ISS particles were more readily observed. This change suggests that replacing a substantial proportion of cement with ISS reduces the clinker content in the composite system, thereby decreasing the formation of major hydration products such as C-S-H gel.

### 3.8. Practical Implications of ISS Utilization

Although ISS remained primarily as residual carbonate phases after hydration, its origin as a water-treatment by-product gives it a resource recovery value distinct from that of natural limestone powder. Using ISS as a partial cement replacement provides a pathway for its utilization in construction materials and may reduce the need for stockpiling and disposal. Its potential use as an alternative source of carbonate powder may also reduce demand for virgin mineral resources. In this study, ISS replacement levels of 5–10% resulted in slightly higher early-age compressive strength and only a small reduction in 28 days strength, providing preliminary performance evidence for its use at low replacement levels. Given the reduction in flowability caused by ISS incorporation at a fixed water-to-binder ratio, future studies could compare mixtures with equal flowability to evaluate hardened properties after workability adjustment. Further comparisons with limestone powder at equal replacement levels and comparable particle size distributions, together with an environmental impact assessment, are needed to clarify the conditions suitable for ISS utilization and its value as a by-product carbonate source.

## 4. Discussion

**Figure 17:**
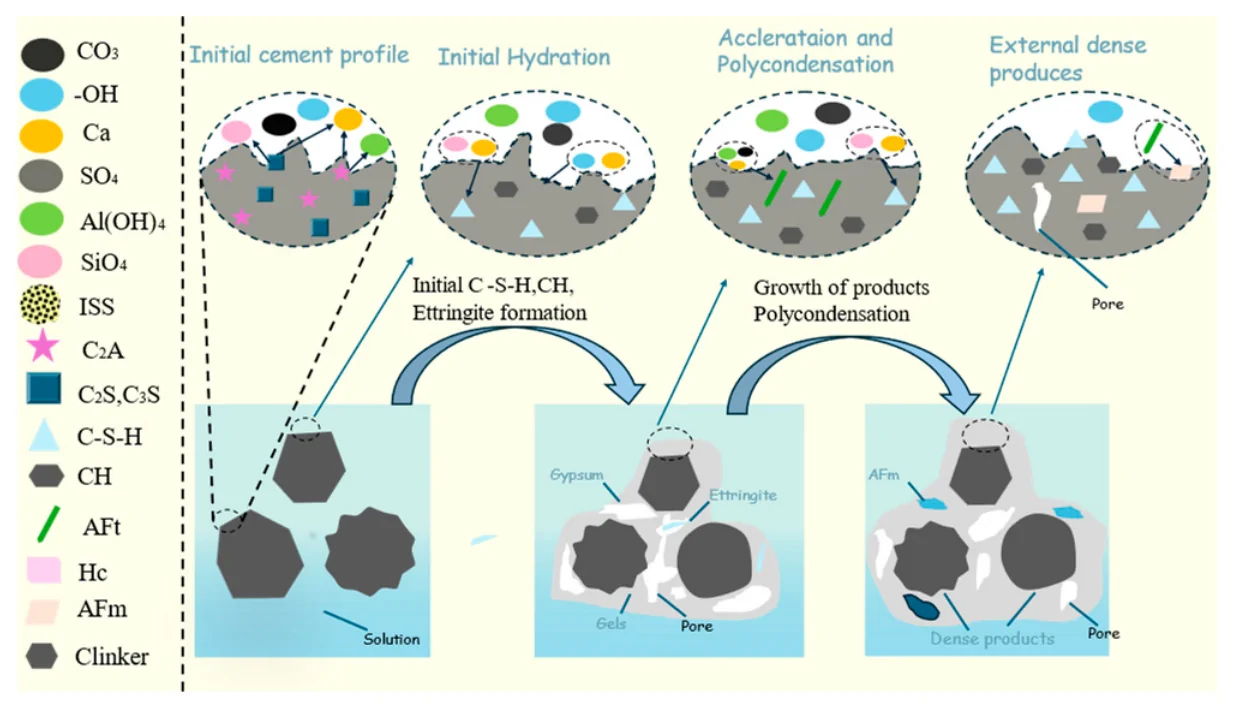
Schematic illustration of the hydration evolution mechanism of the neat OPC system.

**Figure 18:**
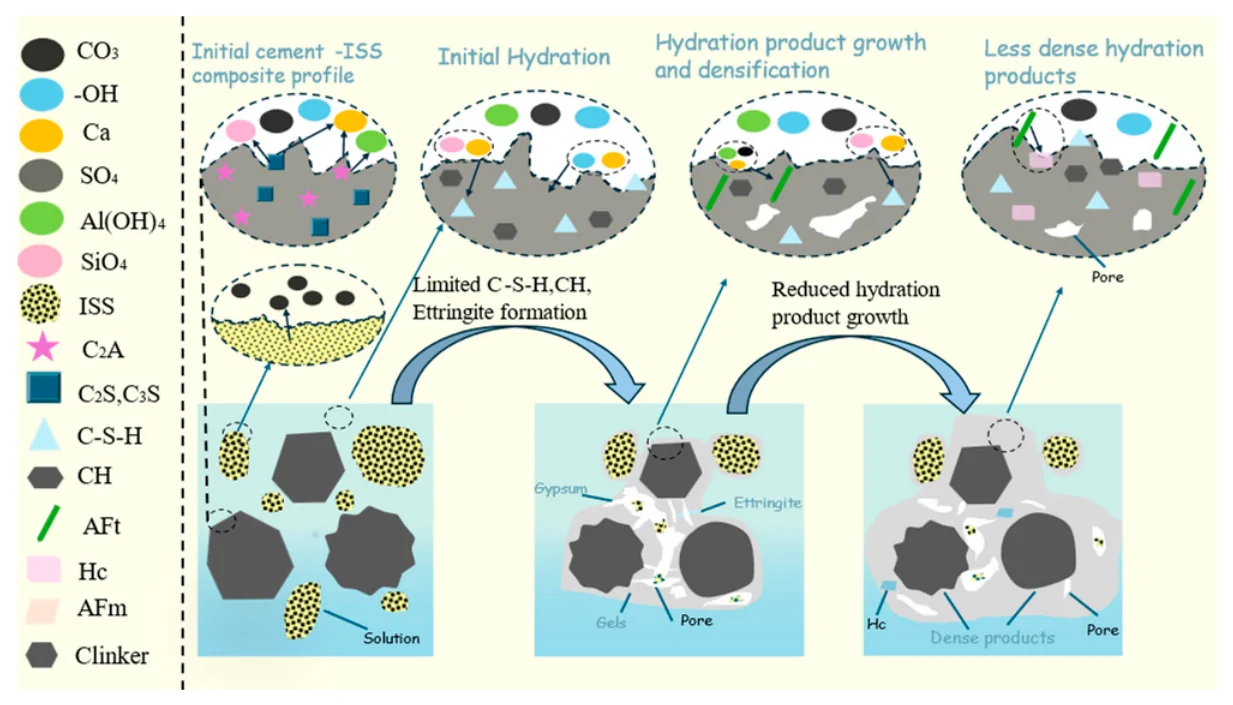
Schematic illustration of the hydration evolution mechanism of the ISS-OPC composite system.

As shown in Figure 17 and Figure 18, ISS incorporation influenced the hydration evolution of cement pastes. In the plain OPC system, clinker particles gradually dissolved, releasing reactive species containing Ca, Si, and Al and forming the main hydration products, including C-S-H gel, CH, and AFt. As hydration proceeded, these products continued to grow and fill interparticle voids, progressively densifying the paste microstructure [44,45]. Since ISS consisted predominantly of calcite (CaCO_3_), which has low solubility under standard curing conditions, its particles remained mainly as residual carbonate particles within the hydrated matrix. ISS had a smaller median particle size than OPC (5.663 versus 13.46 μm). Finer ISS particles may therefore occupy some of the spaces between coarser cement grains, suggesting a potential physical filling contribution. With increasing ISS content, the amount of reactive clinker decreased, limiting the formation of major hydration products such as C-S-H gel and CH. Meanwhile, some of the carbonate introduced by ISS may have participated in the evolution of aluminate-bearing hydrates, promoting hemicarboaluminate (Hc) formation. The Ca- and Si-bearing regions observed around ISS particles also suggest that their surfaces may have provided interfaces for the local deposition of hydration products [46]. At higher ISS replacement levels, the hydrated matrix exhibited reduced local continuity within the fields examined.

The effects of ISS incorporation on the microstructure and strength development of cement pastes involve clinker dilution, possible physical filling and particle surface effects, and carbonate-mediated evolution of hydration phases. At low ISS replacement levels, the smaller median particle size of ISS provides favorable geometric conditions for finer particles to occupy the spaces between coarser cement grains. BSE observations showed that residual particles in the 10% ISS paste were embedded in a relatively continuous hydrated matrix, with Ca- and Si-enriched regions consistent with the composition of C-S-H-like hydration products observed near some particles. These findings are consistent with the possible contribution of fine particles to local microstructural development, which may help maintain interparticle bonding and partially compensate for the adverse effects of clinker replacement. This interpretation is consistent with the limited loss of 28 days compressive strength at low ISS replacement levels. Combined with the early-age changes in CH observed by XRD and TG analyses, the physical effects of ISS particles and changes in hydration behavior may have jointly contributed to the slight increase in early-age compressive strength.

In contrast, the reduced local continuity of the hydrated matrix observed in the 20% ISS paste may be unfavorable for interparticle bonding, consistent with the more pronounced loss of later-age strength. Therefore, physical filling may be an important potential mechanism contributing to strength retention at low ISS replacement levels, whereas the Ca- and Si-enriched regions surrounding ISS particles only suggest the local deposition of hydration products.

## 5. Conclusions

This study systematically investigated the effects of ISS incorporation on the macroscopic properties and microstructural evolution of cement-based materials. The findings provide a basis for understanding the role of ISS in cement hydration and demonstrate its potential for resource utilization in construction materials.

1. In terms of mechanical performance, a replacement level of 5–10% ISS is recommended when ISS is used to partially replace cement. An appropriate ISS content can improve the early-age compressive strength of the composite paste, whereas excessive ISS incorporation results in a pronounced loss in later-age strength.
2. In terms of fresh-state properties, the incorporation of ISS reduced the flow diameter and prolonged the setting time of the cement paste. With increasing ISS content, the flow diameter gradually decreased, while both the initial and final setting times were continuously prolonged.
3. Regarding hydration products, ISS incorporation did not appreciably alter the types of major hydration products in the system. Calcite introduced by ISS remained detectable after hydration, while the identification of hemicarboaluminate (Hc) in the ISS-containing pastes suggests that carbonate species may have participated in the evolution of aluminate-bearing hydrates.
4. Regarding microstructural morphology, BSE/EDS observations revealed residual ISS-associated particles distributed within the hydrated matrix. At higher replacement levels, the matrix exhibited reduced local continuity within the fields examined, consistent with the decline in later-age compressive strength.

## Figures and Tables

**Table 1:** Chemical composition of the raw materials.

Composition (%)	SiO_2_	Al_2_O_3_	Fe_2_O_3_	CaO	MgO	K_2_O	Na_2_O	SO_3_	P_2_O_5_	Cl	Cu	Zn
OPC	18.80	5.68	3.38	63.59	1.78	1.07	0.211	4.78	0.0485	-	-	-
ISS	9.331	0.519	1.366	70.73	10.91	0.081	1.57	3.83	0.106	0.315	0.0182	0.0252

**Table 2:** Mix proportions of the composite pastes.

Group	OPC-42.5 (wt%)	ISS (wt%)	w/b
I0	100	0	0.4
I5	95	5	
I10	90	10	
I15	85	15	
I20	80	20	
I25	75	25	
I30	70	30	

**Table 3:** Mass loss of the composite pastes in different temperature ranges at 3 days.

Temperature	50–200 °C	400–500 °C	600–800 °C
OPC-3 days	4.133%	2.893%	2.98818%
10%-3 days	3.765%	3.028%	4.3854%
20%-3 days	3.893%	2.776%	6.291%
30%-3 days	3.807%	2.889%	8.101%

**Table 4:** Mass loss of the composite pastes in different temperature ranges at 28 days.

Temperature	50–200 °C	400–500 °C	600–800 °C
OPC-28 days	10.17543%	3.88828%	3.5506%
10%-28 days	7.9661%	3.25625%	4.74409%
20%-28 days	6.71545%	3.08998%	7.45665%
30%-28 days	7.52045%	3.23447%	10.0298%

## Data Availability

The original contributions presented in this study are included in the article. Further inquiries can be directed to the corresponding author.
